# Introduction to the Special Issue on *Advances in Biological Tissue Biomechanics*

**DOI:** 10.3390/bioengineering7030095

**Published:** 2020-08-17

**Authors:** Chung-Hao Lee, Jun Liao

**Affiliations:** 1 Biomechanics and Biomaterials Design Laboratory (BBDL), School of Aerospace and Mechanical Engineering, The University of Oklahoma, Norman, OK 73019, USA; 2 Institute for Biomedical Engineering, Science and Technology (IBEST), The University of Oklahoma, Norman, OK 73019, USA; 3 Tissue Biomechanics and Bioengineering Laboratory (TBBL), Department of Bioengineering, University of Texas at Arlington, Arlington, TX 76010, USA

**Keywords:** in vivo stress/strain quantification, constitutive models, soft tissues, growth and remodeling (G & R), multiscale biomechanics, patient-specific modeling, biomechanics

## 1. Introduction

Advanced experimental and computational biomechanics have become essential components to better understand the physiological and pathological conditions of biological tissues in the human body. Recent advances in medical imaging modalities, image segmentation, tissue characterization experiments, and predictive computer simulations have made major contributions to transforming current therapeutic paradigms towards the facilitation of patient-specific diagnostics and individualized surgery planning. This *Bioengineering* special issue on *Advances in Biological Tissue Biomechanics*, therefore, focuses on research dealing with cutting-edge experimental and computational methodologies for biomechanical investigations of tissues in the human body system across multiple spatial and temporal scales.

## 2. Individual Contributions

### 2.1. Experimental Characterizations and Computational Modeling of Cardiac Heart Valves

Valvular heart disease (VHD) encompasses a number of common cardiovascular conditions that account for 10 to 20% of all cardiac surgical procedures in the United States [1]. A better understanding of the natural history coupled with the major advances in diagnostic imaging, interventional cardiology, and surgical approaches have resulted in accurate diagnosis and appropriate patient–treatment matching for therapeutic interventions. A thorough understanding of the various valvular disorders is important to aid in the management of patients with VHD, especially as the U.S. population ages [2]. This special issue collects biomechanical studies of the tricuspid valve (TV), the aortic valve (AV), and mechanical heart valve replacement devices via groundbreaking experimental and computational technologies.

Lee et al. (2019) [3] provided a comprehensive review (the first of its kind) on the current diagnoses and treatments for functional tricuspid regurgitation from the clinical perspective, the state of the art for in-vivo and in-vitro biomechanical investigations of the subvalvular components of the tricuspid heart valve (e.g., TV annular dynamics, biaxial mechanical properties of the TV leaflets, pressure-induced changes in the collagen fiber organization, and TV chordae tendineae mechanics), and advanced in-silico biomechanical modeling techniques for the TV, including isogeometric analysis-based geometry construction, tissue-structure-informed constitutive model developments, and bio-solid and fluid-structure interaction simulations of the TV closing behaviors. Potential future research directions and developments of innovative technologies for improved management of functional tricuspid regurgitation were also discussed.

Novel multiscale characterizations for the three TV leaflets were presented by Hudson et al. (2020) [4]. In this pilot study, Hudson et al. used an integrated experimental approach, which combined a biaxial testing apparatus with a polarized spatial frequency domain imaging device, to observe the load-dependent changes in the collagen fiber architectures (CFAs) for the three TV leaflets from representative porcine hearts. Their results from this pilot study could provide key insight into the connections between the microstructure and tissue mechanics of the TV. The methods employed in their study also show promise for future works to investigate the load-dependent changes in diseased TV leaflets’ CFAs, which would provide a better understanding of the effects of disease on the microstructure–mechanics relationships of the cardiac heart valve tissues and help inform TV computational models for guiding clinical therapeutics, for example, in terms of the feasibility or effectiveness of valve repair techniques.

In soft tissue biomechanics, characterizations of a tissue’s mechanical properties have traditionally been facilitated through bench-top experiments, such as uniaxial or biaxial tensile extension tests, in which tissue samples are immersed in a buffer solution (e.g., phosphate buffered saline (PBS)). Salinas et al. (2019) [5] conducted an in-vitro experiment to examine whether maintaining the physiological osmolarity of the tissue may alter the mechanical response of the tricuspid valve (TV) anterior leaflet. Their study demonstrated the influence of a hypo-osmotic environment on the quantified mechanical behaviors of TV anterior leaflet tissue. The imbalance in ions leads to water absorption in the valvular tissue that can alter its mechanical responses. As such, it is essential to use isotonic buffer solution in ex-vivo experiments to characterize the mechanical properties of native valve tissues.Their work provided quantitative justification for the use of isotonic buffers and will provide a valuable citation in guiding future studies of biomechanical characterizations of tricuspid valve tissue.

Next, Ross et al. (2020) [6] reviewed the current status of tissue biomechanics for the chordae tendineae of the atrioventricular heart valves (AHVs), i.e., the mitral and tricuspid valves. Besides a comprehensive summary of the existing biomechanical characterizations and microstructural quantifications of the AHVs’ chordae tendineae, Ross et al. also presented novel results from their studies [7] on the chordae-leaflet insertion for the chordae tissues. In this work, the coupled polarized spatial frequency domain imaging and uniaxial testing method (similar to the integrated approach in Hudson et al. [4]) was used to analyze the changes in the load-dependent collagen fiber orientations of the strut chordae insertions of porcine AHV anterior leaflets under varied uniaxial tensile loads. Ross et al. concluded this review article by summarizing perspectives for future studies that could help better understand the mechanics–microstructure linking of chordae tendineae tissues and improve the therapeutics for valvular heart diseases caused by chordae failure.

The progression of calcific aortic valve disease (CAVD) is the consequence of extracellular matrix (ECM) remodeling, leading to structural abnormalities and improper valve function. Tesfamariam et al. (2019) [8] provided insight to the effects of ECM remodeling on the aortic valve leaflet axial curvature changes by performing elastin degradation via enzymatic digestion. They found that the elastin-degraded leaflets had significant increases in the mean curvature as compared to the undegraded (control) specimens, while this significant change was not observed when the measurements were performed using either the minimum or maximum curvature. These results suggest that the mean axial curvature metric can be used to detect distinct spatial changes in the aortic valve ECM arising from the loss in the bulk content and/or structure of elastin. Therefore, the instance of maximum leaflet flexure during the cardiac cycle could be targeted for the mean curvature measurements, serving as a potential biomarker for elastin degradation in early CAVD remodeling.

Artificial heart valves may become dysfunctional, leading to thrombus and/or pannus formations. Computational fluid dynamics (CFD) is a promising tool for improved understanding of heart valve hemodynamics, allowing quantification of detailed flow velocities and turbulent stresses to complement in-vivo clinical Doppler measurements. Khalili et al. (2018) [9] investigated the flow characteristics and hemodynamic performance of a bileaflet mechanical heart valve via CFD simulations with a more realistic aortic sinus geometry than was used previously in similar works. The modeling results of this study suggested that higher levels of valve dysfunction are accompanied with flow separation at the leaflet surfaces and growing eddies, especially downstream of the valve in the aortic sinuses. Additionally, the principal turbulent stresses for immobile leaflets could exceed the threshold values for elevated risk of hemolysis and platelet activation, further leading to potential development of thrombosis, especially around the normal leaflet. This computational modeling approach may be adapted to serve as a patient-specific tool to identify adverse conditions that are associated with an increased risk of hemolysis and thrombus formation, capturing a more complete picture of the valve status in clinical management of patients with dysfunctional valves.

### 2.2. Biomechanical Investigations of Cardiovascular/Vascular Tissues

The etiology of abdominal aortic aneurysm (AAA) development is believed to be multi-factorial, in that the pathology is initiated at the molecular level (protease- and enzyme-related), builds up to the tissue level through extracellular matrix (ECM) and structural changes, and manifests as geometrical-, biomechanical-, and blood-flow-related alterations in the abdominal aorta. Of the numerous etiological theories of AAA pathology, the degraded ECM theory is most widely accepted, as human AAA specimens usually exhibit a reduction in elastin content and an increase in collagen cross-linking [10]. AAA porcine models based on elastase–collagenase combination treatments are uncommon, but their combined effects produce maximal damage to the ECM and pronounced in-vivo inflammatory infiltration. In this work, Patnaik et al. [11] quantified pentagalloyl glucose (PGG)-mediated biomechanical restoration of degenerated ECM, which serves as a “proof-of-concept” investigation of the cross-linking properties of PGG specificality with respect to the degenerated arterial ECM. In other words, PGG leads to the cross-linking between the ECM proteins, improving the biomechanical strength of enzymatically degraded tissues. The primary contributions of this work are the quantification of the biomechanical restoration potential of PGG and its relation to the PGG binding to arterial ECM.

Despite the advances in modern clinical management, heart failure (HF) maintains a high mortality and morbidity in the United States. More than 5 million Americans have HF, and around 550,000 new cases occur every year [12]. Liu and Wang [13] reviewed the current understanding of the biomechanics of ventricular tissues. They also presented the common methods for characterizations of the ventricles, the known ventricular mechanical properties including the viscoelasticity of the tissue, the existing computational models, and the clinical relevance of the ventricular mechanical properties. Furthermore, Liu and Wang suggested future research directions, including characterizing the viscoelastic properties of the ventricles at different physiological and pathological conditions and examining how the acellular and cellular components affect the ventricular tissue’s viscoelastic properties. These future research efforts will elucidate the roles of ventricular biomechanics in ventricular dysfunction, enhancing current insights of the pathogenesis of RV failure or biventricular failure and to inspire new therapies for patients with heart failure.

### 2.3. Hydration Effect on the Compressive Tissue Mechanics of Brain

Traumatic brain injury (TBI), primarily induced by mechanical impact to the head, is a leading cause of death and life-long disability in the United States. Around 5.3 million Americans currently have long-term disabilities after sustaining a TBI [14]. Designing protective systems for the human head and the brain requires a better fundamental understanding of the brain’s microstructural response to mechanical insults. Prabhu et al. [15] assessed the effects of hydration on the mechanical behavior of porcine brain over a range of strain rates (quasi-static and high dynamic rates) and complemented these results with an analysis of tissue hydration effects using finite element analyses (FEAs) at high strain rates. Their experimental results showed a strong strain rate dependence for the wet brain (∼80% m/m), whereas the dry brain’s tangent modulus, elastic–inelastic transition stress, and the strain at yield point were strain-rate insensitive. In addition, micromechanical FEA considering various proportions of water in the dry brain further demonstrated that water plays a major role in the initial hardening trend of the brain’s biomechanical behaviors. These novel results highlight the importance of incorporating the hydration effect into simulations of the brain’s mechanical responses under injury scenarios or virtual human-centric protective headgear design.

### 2.4. High Strain Rate Mechanical Responses of Liver Tissue

In automobile accidents, abdominal injuries are often life-threatening yet not apparent at the time of initial injury. The liver is the most commonly injured abdominal organ from this type of trauma owing to its fragile material properties. Efforts to determine the optimal safety measures for automobile-related accidents have largely relied on crash dummies, which exhibit significant limitations in recapitulating injury impact to humans. Since the 1970s, there have been no substantial changes in assessing injury. In this study, Chen et al. [16] utilized a polymeric split-Hopkinson pressure bar (PSHPB) testing system to explore the effects of strain rates on the material behaviors of porcine liver tissue. Three key findings from this study are: (i) the liver tissue response at high-rate compression was characterized by an initial hardening peak, followed by softening, and then by strain hardening to failure; (ii) the liver’s mechanical stiffness increased as the applied strain rate increased; and (iii) an isotropic high-rate material behavior was observed along all three orthogonal directions and was confirmed by the liver histological microstructure. Experimental data of this work coupled with finite element modeling can be implemented in large-scale simulations of the human body considering extreme, high-strain-rate scenarios, such as automobile accidents, to guide automobile safety measures that will reduce the risk of abdominal injuries in high-impact situations.

### 2.5. Linking Tissue Mechanics and Extracellular Matrix for Venous Valve Tissue

Jugular venous valve incompetence has no long-term remedy and symptoms of transient global amnesia and/or intracranial hypertension continue to discomfort patients. Benson and Huang [17] examined the synergy of the collagen and elastin microstructure that compose the bi-layer extracellular matrix (ECM) of the jugular venous valve. In this study, Benson and Huang investigated the jugular venous valve and related the tissue-level mechanical properties, fibril orientation, and fibril composition. Light microscopy was used to deduce that the venous valve’s isolated elastin microstructure was unaffected by their drying methods. In addition, light microscopy of the collagen microstructure allowed characterizations of the crimp effects on the venous valve’s mechanical properties, indicating that collagen aligning circumferentially and elastin orienting radially attributing to a stiffer response in the circumferential direction. Force-controlled mechanical testing also provided the proof that the elastin’s cross-linked mesh accounts for the circumferential direction’s mechanical properties at low strains. In short, this work outlined the contribution of both the collagen and elastin microstructures to the physiological function of the jugular venous valve tissue. This new knowledge of the venous valve tissue-level microstructures is important for advances in basic venous physiology and for future novel approaches to prevent venous valve incompetence.

### 2.6. Key Considerations for Biomechanical Parameter Estimations of Soft Tissues

Several nonlinear and anisotropic constitutive models have been proposed to describe the biomechanical properties of soft tissues, and reliably estimating the unknown parameters in these models using experimental data is an important step towards developing predictive capabilities. However, the effect of parameter estimation techniques on the resulting biomechanical parameters remains under-analyzed. Aggarwal [18] conducted a thorough study to to analyze the effects of parameter estimation on biomechanical characterizations of soft tissues under planar biaxial testing, including nonlinear preconditioning of the data, selection of the weighted residual, inclusion of the fiber angle as an estimation parameter, and displacement-controlled versus force-controlled testing. Specifically, four invariant-based constitutive models for soft tissues were tested: the Gasser–Ogden–Holzapfel (GOH) model [19], the Humphrey model [20], the Lee-Sacks model [21], and the May–Newman model [22], each with their own set of five or six parameters. It was found that small modifications of weighting the residual by the experimental data and/or taking a log of the parameter in front of the exponential can significantly improve the parameter estimation process. The advantages of the proposed modifications were found not only in terms of convergence speed but also in reducing the possibility of estimating wrong parameter values by getting stuck in a local minima. These results suggest that determining the fiber angles using a non-mechanical test, as in, for example, an optical technique, can greatly help the parameter estimation process.

## 3. Conclusions

Altogether, the 12 research papers/reviews in this Special Issue on *Advances in Biological Tissue Biomechanics* reflect the importance of experimental and computational biomechanics for improving the fundamental understanding of the function and properties of biological tissues in human body systems. These essential research investigations and technological developments will serve as a foundation for future advances in precision medicine for accurate diagnosis and effective prophylactic management. Finally, the guest editors would like to thank all the authors for their appreciated contributions.
